# A Low-Power and Robust Micromachined Thermal Convective Accelerometer

**DOI:** 10.3390/mi15070844

**Published:** 2024-06-29

**Authors:** Yizhou Ye, Shu Wan, Chen Hou, Xuefeng He, Shunbo Li

**Affiliations:** Key Laboratory of Optoelectronic Technology and Systems, Ministry of Education, Chongqing University, Chongqing 400044, China; yzye@cqu.edu.cn (Y.Y.); wanshu@cqu.edu.cn (S.W.); chenh89@cqu.edu.cn (C.H.); hexuefeng@cqu.edu.cn (X.H.)

**Keywords:** accelerometer, thermal convection, MEMS, tilt sensor

## Abstract

This paper presents a micromachined thermal convective accelerometer with low power and high reliability. This accelerometer comprises a heater and two thermistors. The central heater elevates the temperature of the chip above ambient levels while the symmetrically arranged thermistors monitor the temperature differentials induced by acceleration. The heater and thermistors are fabricated on a glass substrate using a standard micro-electromechanical systems (MEMS) process flow, and the fabricated sensor is installed on a rotation platform and a shaking table experimental setup to perform the experiment. The results indicate that the sensor has the capability to measure accelerations surpassing 80 m/s^2^, with an approximate linear sensitivity of 110.69 mV/g. This proposed thermal convective accelerometer offers promising potential for various applications requiring precise acceleration measurements in environments where low power consumption and high reliability are paramount.

## 1. Introduction

Accelerometers have become indispensable sensors across a variety of fields, spanning the automotive industry to consumer electronics, the aerospace industry, structural health monitoring, industrial automation, healthcare, and robotics [1,2,3,4,5]. In recent years, the rapid advancement of semiconductor manufacturing has dramatically accelerated the development of accelerometers utilizing MEMS technology. Various types of accelerometers based on different principles, such as resonant [6,7,8,9,10], capacitive [11,12,13], thermal convection [14,15,16,17,18], and optical [19,20,21,22,23], have been successfully fabricated [6,7,8,9,10,11,12,13,14,15,16,17,18,19,20,21,22,23]. Among these, MEMS accelerometers employing thermal convection have emerged as a significant branch due to their notable advantages, which include a straightforward structure, compact dimensions, and seamless integration with conditioning circuits. However, during the operation of the thermal convective accelerometer, it is necessary to heat the sensor chip to a temperature higher than the ambient temperature. Silicon, a material with high thermal conductivity, efficiently dissipates heat through the silicon substrate. As a result, thermal convective accelerometers fabricated on silicon substrates typically require significant power consumption to maintain the desired operating temperature.

To address the issue of spurious heat dissipation through the silicon substrate and to minimize sensor power consumption, conventional MEMS thermal convective accelerometers typically employ suspended configurations for the heater and thermistors, either on a membrane or elevated above the substrate [24,25,26,27]. However, these suspended structures are vulnerable to intense vibrations or mechanical shocks [28,29]. Additionally, fluctuations in thermal stress resulting from temperature changes can detrimentally impact sensor performance [28,30,31].

The inherent drawbacks of the suspension membranes and the high power consumption associated with the silicon substrates emphasize the need for a thermal convective accelerometer that balances structural robustness and energy efficiency. Therefore, glass with low thermal conductivity has become the preferred substrate material for fabricating thermal convective accelerometers. Incorporating a glass substrate holds promise for delivering two significant benefits to the MEMS thermal convective accelerometer. Firstly, owing to the notably lower thermal conductivity of glass compared to silicon, positioning the heater and the thermistors on the glass substrate leads to a marked decrease in the dissipation of ineffective heat through the substrate. Moreover, the low thermal conductivity of the glass substrate significantly reduces the necessity of processing the substrate into suspended structures. Directly placing the heater and the thermistors onto the bulk glass allows the sensor to achieve lower power consumption. Thus, adopting a low thermal conductivity glass substrate enables the development of low-power and highly reliable thermal convective accelerometers.

In previous work, our group utilized a glass reflow process to fabricate a silicon-in-glass (SIG) substrate, which was then employed to prepare a thermal convective accelerometer [32]. Although this approach significantly reduces power consumption compared to silicon-based devices, the substrate stress induced by the glass reflow process leads to significant performance variations across sensors fabricated on this substrate, resulting in extremely low yield rates. Therefore, in this paper, the commercially available glass wafer Pyrex7740 is utilized to fabricate the thermal convective accelerometers.

## 2. Principle and Analysis

**Figure 1:**
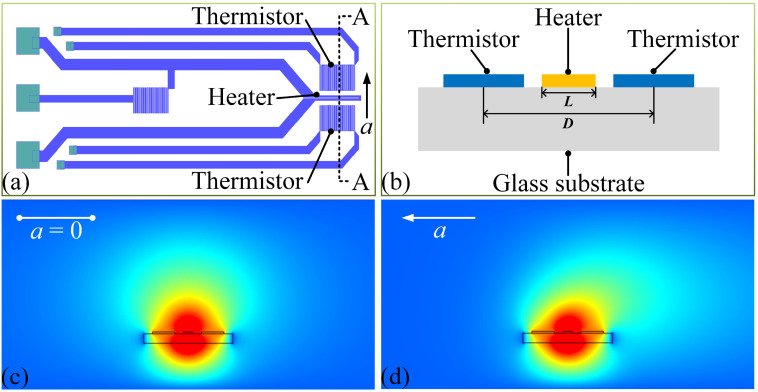
(**a**) Schematic overview of the MEMS thermal convective accelerometer. (**b**) A-A sectional view of the thermal convective accelerometer. (**c**) Temperature distribution on the surface of the sensor chip in the absence of acceleration. (**d**) Temperature distribution on the surface of the sensor chip when a leftward acceleration is applied.

The structure and the operational principle of the MEMS thermal convective accelerometer are elucidated in Figure 1. The accelerometer features a central heater with two thermistors positioned symmetrically on either side, all arranged in a serpentine configuration. The heater is located at the center of the chip, while the thermistors are equidistant on either side of the heater. Figure 1b provides the A-A sectional view of the active area of the chip. When the sensor is not subjected to acceleration, the thermal distribution across the chip surface remains symmetrical, with both thermistors registering the same temperature, as depicted in Figure 1c. However, when an acceleration is applied to the sensor, this thermal symmetry on the chip surface is disrupted, causing the two thermistors to record different temperatures, as shown in Figure 1d. At this time, the acceleration can be determined by measuring the temperature differential between the two thermistors.

For MEMS sensors operating on the thermal principle, high power consumption poses a significant limitation. Typically, the total power dissipation *P_all_* of a thermal sensor consists of conductive heat transfer *P_d_* and convective heat transfer *P_v_*, with thermal radiation often considered negligible. Hence, the power consumption *P_all_* of the sensor can be mathematically represented as

(1) $$P_{all}=P_{d}+P_{v}$$

In a thermal convective accelerometer, the heater generates Joule heat as electrical current flows through it, causing an increase in temperature. This heat is then conducted from the heater to the substrate through their interface and dissipated into the surrounding air through convection. Since thermal conduction is generally more efficient at dissipating heat than thermal convection, and acceleration measurement in these sensors relies on detecting changes in thermal convection, it is critical to optimize heat exchange with the surrounding air while minimizing heat conduction from the heater to the substrate.

Various methods have been explored to alleviate thermal conduction between the heater and substrate and further reduce the power consumption of the sensor. These methods include positioning the heater and thermistors on suspended membranes or elevating them above the substrate [24,25,26,27]. However, the constructed suspended structures often lack resilience against vibrations or shocks [28,29], and thermal stress variations caused by temperature changes can negatively affect sensor performance [28,30,31].

To address these challenges, this paper proposes the adoption of a glass substrate with low thermal conductivity to fabricate the MEMS thermal convective accelerometer. The utilization of the glass can effectively reduce inefficient energy dissipation through the substrate thermal conduction, allowing more heat to be available for exchange with the surrounding airflow.

In the convective heat transfer principle, when acceleration is applied along the direction connecting the two thermistors, the resulting temperature difference between the two thermistors is directly proportional to the Rayleigh number *Ra* [33]. The Rayleigh number represents the ratio of inertial force to viscous force and is commonly employed to characterize convection flow within the chip. Thus, the temperature difference *S* between the two thermistors on the chip surface can be expressed by [34,35,36]

(2) $$S\propto Ra=\frac{\beta C_{p}\rho^{2}L^{3}\Delta T}{\mu k}a$$

Here, *β*, *C*_p_, *ρ*, *μ*, and *k* are coefficient of fluid expansion, specific heat, density, dynamic viscosity, and thermal conductivity of the fluid, respectively. *L* is the width of the heater, Δ*T* is the overheat temperature of the heater to the ambient, and *a* denotes the applied acceleration.

This equation suggests that if the fluid property parameters (*β*, *C*_p_, *ρ*, *μ*, and *k*), the overheating temperature (Δ*T*), and the heater width (*L*) remain constant despite temperature fluctuations, the resulting temperature difference *S* between the two thermistors will solely depend on the applied acceleration *a* [37]. Consequently, determining acceleration becomes feasible by measuring the temperature difference between the two thermistors.

## 3. Experiment and Discussion

**Figure 2:**
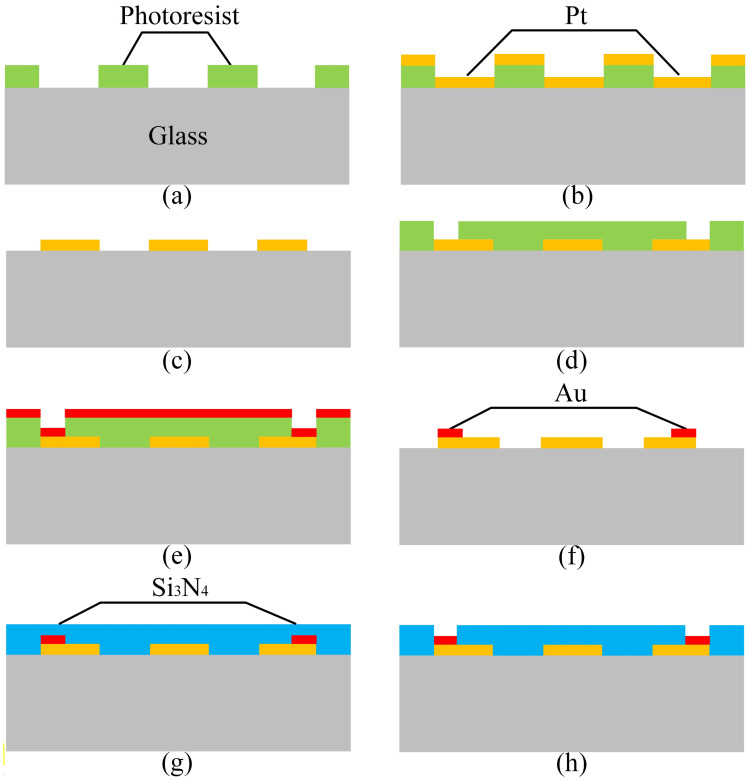
The fabrication process of the MEMS thermal convective accelerometer. (**a**) Patterning on the glass wafer. (**b**) Depositing Pt film. (**c**) Lifting off to create the heater and thermistors. (**d**) Patterning on the glass wafer. (**e**) Depositing Au film. (**f**) Lifting off to define the pads for wire bonding. (**g**) Depositing Si_3_N_4_ to form the passivation layer. (**h**) Etching the passivation layer to expose the pads.

The proposed MEMS thermal convective accelerometer is fabricated using a standard MEMS process. The fabrication process, illustrated in Figure 2, consists of eight main steps: (1) patterning on the glass wafer, (2) deposition of Pt film, (3) lift-off for creating the heater and thermistors, (4) additional patterning on the glass wafer, (5) deposition of Au film, (6) lift-off to define wire bonding pads, (7) deposition of Si_3_N_4_ to form the passivation layer, and (8) etching the passivation layer to expose the pads.

To balance power consumption and robustness, a double-side-polished 4-inch Pyrex7740 glass wafer with a thickness of 500 μm serves as the substrate. Positive photoresist AZ5214, 2 μm thick, is spin-coated and patterned via photolithography onto the glass wafer. Subsequently, a sputtering process deposits a 150 nm-thick Pt film. To enhance Pt adhesion, a 30 nm Ti interlayer is utilized. After sputtering, the wafer is soaked in acetone to lift off redundant Pt, leaving the heater and thermistors on the substrate. Following this, a 300 nm-thick Au film is deposited and patterned onto the substrate via the lift-off process to create pads for wire bonding. Then, a low-stress 300 nm Si_3_N_4_ film is deposited using a low-temperature plasma-enhanced chemical vapor deposition (PECVD) process to form a passivation layer for the sensor. This passivation layer prevents direct exposure of the active elements to harsh media, enhancing sensor reliability. Finally, reactive ion etching (RIE) removes the Si_3_N_4_ film above the pads, exposing them for wire bonding.

**Figure 3:**
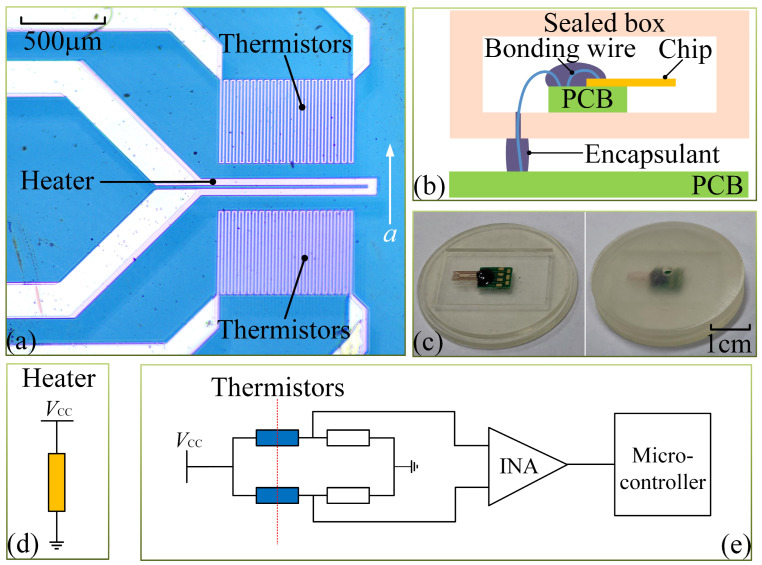
(**a**) Photograph of the fabricated MEMS thermal convective accelerometer. (**b**) The schematic diagram of the sensor packaging. (**c**) Photographs of the sensor chip housed inside the sealed box. (**d**) The schematic of the interface circuit for the heater. (**e**) The schematic of the interface circuit for the thermistors.

Figure 3a presents the photographs of the fabricated MEMS thermal convective accelerometer, which measures 3 mm × 8 mm × 0.5 mm in size. The resistances of the fabricated heater and thermistors were measured at 72 Ω and 1.5 kΩ, respectively, at room temperature, with a temperature coefficient of about 3236 ppm/K within the temperature range of −10 °C to 40 °C.

**Figure 4:**
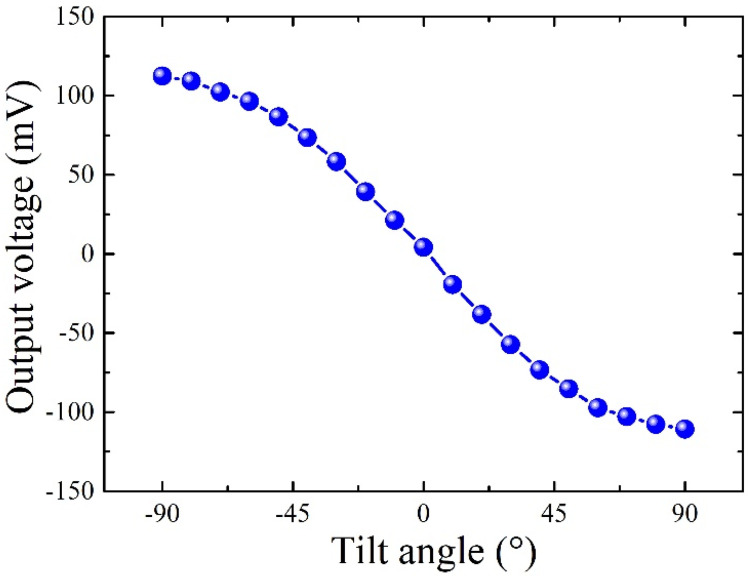
The tilt angle test results of the MEMS thermal convective accelerometer when the sensor rotates from +90° to −90° around the central axis of the heater.

The fabricated accelerometer chip was connected via leads to a printed circuit board (PCB) and subsequently placed within a sealed box fabricated using 3D printing technology, achieving hermetic packaging. The overall schematic diagram of the packaging structure is depicted in Figure 3b, and Figure 3c shows the photograph of the chip housed inside the sealed box. Moreover, a PCB interface circuit is employed to evaluate the performance of the MEMS thermal convective accelerometer. In this setup, a constant voltage heats the central heater, depicted in Figure 3d, while a Wheatstone bridge is formed using two fixed resistances and the two thermistors on the chip, as shown in Figure 3e. This sensor system was then mounted onto a rotation platform to assess its response to various tilt angles. During the experiment, the heater delivered approximately 120 mW of driving power. When the sensor rotates along the central axis of the heater, the voltage output of thermistors is shown in Figure 4. The proposed accelerometer exhibits remarkable acumen in discerning inclination angles from +90° to −90°. The measurement sensitivity of the accelerometer is quantified at 110.69 mV/g.

**Figure 5:**
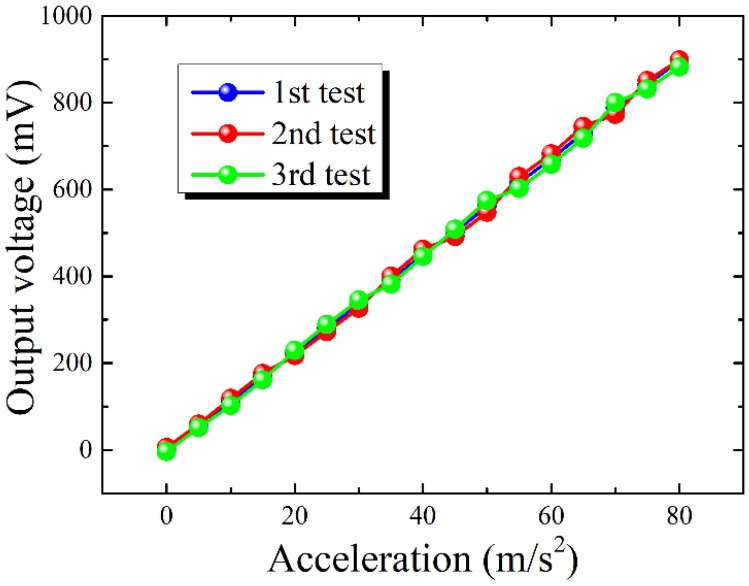
The test results of the horizontally positioned MEMS thermal convective accelerometer when subjected to acceleration perpendicular to the central axis of the heater.

In addition, the accelerometer was placed horizontally, and subjected to acceleration perpendicular to the central axis of the heater. The experimental shaking table setup was used to measure the output voltage variation of the accelerometer at different accelerations. The experiment was repeated three times on the same chip with an input acceleration range of 0 to 80 m/s^2^, and the corresponding results are shown in Figure 5. It can be observed that the sensor is capable of measuring accelerations exceeding 80 m/s^2^. Within this measurement span, the output voltage of the device exhibits almost linear variation with increasing input acceleration and demonstrates commendable repeatability.

**Figure 6:**
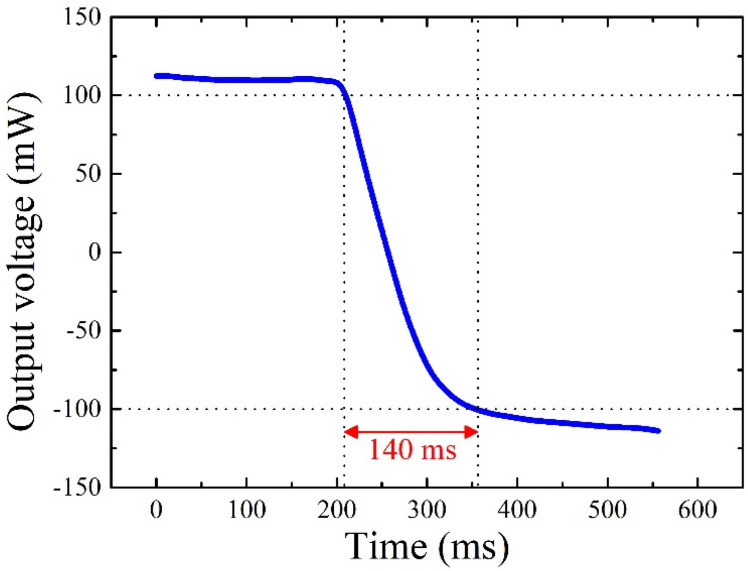
The response time testing results of the MEMS thermal convective accelerometer.

Furthermore, the response time of the thermal convective accelerometer was evaluated by activating the heater in constant voltage (CV) mode. During this evaluation, the sensor was subjected to rotational maneuvers around the central axis of the heater, inducing an abrupt shift in tilt angle from +90° to −90°. The microcontroller recorded the subsequent variations in output voltage, meticulously portrayed in Figure 6. In this context, the response time signifies the duration necessary for the output voltage to transition from 90% to 10% of its maximum value as the tilt angle oscillates from +90° to −90°. Notably, the data delineate a response time of approximately 140 ms, underscoring the swift and precise responsiveness of the sensor to dynamic environmental changes.

## 4. Conclusions

This paper presents the fabrication and characterization of a micromachined thermal convective accelerometer designed for low power consumption and high reliability. Fabricated on a glass substrate, the sensor leverages low thermal conductivity glass to minimize heat conduction between the heater and substrate, thus reducing power consumption. Additionally, low thermal conductivity glass eliminates the need to hollow out the substrate into a suspended structure, further enhancing sensor reliability. The fabricated thermal convective accelerometer is installed on a rotation platform and a shaking table experimental setup for the experiment. The results indicate that the sensor has the capability to measure accelerations surpassing 80 m/s^2^, with an approximate linear sensitivity of about 110 mV/g. The proposed thermal convective accelerometer holds significant promise for applications requiring precise acceleration measurements in environments prioritizing low power consumption and high reliability.

## Data Availability

The data presented in this study are available on request from the corresponding author.
